# Sensorless Payload Estimation in an Industrial Robot Using Internal Motor-Current Signals

**DOI:** 10.3390/s26154867

**Published:** 2026-08-02

**Authors:** Adam Bátrla, Ojan Majidzadeh Gorjani, Radek Byrtus, Radim Hercík

**Affiliations:** Department of Cybernetics and Biomedical Engineering, Faculty of Electrical Engineering and Computer Science, VSB—Technical University of Ostrava, 708 00 Ostrava, Czech Republic

**Keywords:** virtual sensing, motor-current signature analysis, statistical feature extraction, multilayer perceptron, cross-robot generalization, unit-to-unit variability, payload verification

## Abstract

This article presents a sensorless method for cycle-level payload mass estimation in industrial robots using internal motor-current signals only. The approach is based on automated KUKA Trace acquisition of six-axis current traces from a KUKA KR3 controller, followed by statistical feature extraction and regression using a multilayer perceptron (MLP). Experiments were conducted on two nominally identical KUKA KR3 R540 manipulators under a repeatable handling motion with payloads ranging from approximately 0.4 kg to 2.6 kg, enabling the systematic engineering validation of controller-based current acquisition, feature-based payload regression, and cross-robot validation. This study investigated whether motor-current signals could serve as a reliable virtual sensing source without external force or weight sensors. Under repeated operating conditions, the most accurate MLP configuration achieved a testing mean absolute error (MAE) of 6.75 g and a mean squared error (MSE) of 68.28 g^2^. With mixed-source training, using data from both robots, the testing MAE decreased to 5.37 g, and the accuracy within a ±15 g tolerance reached 96.88%. In contrast, direct transfer to another nominally identical robot increased the MAE to 80.54 g, revealing a clear cross-robot generalization gap. Overall, this study demonstrates the feasibility of motor-current-based payload verification during repeated single-axis motion under controlled conditions and indicates its potential for gripping validation and missing-part detection in industrial handling applications.

## 1. Introduction

The ability of industrial robots to monitor their own operational state in real time is becoming increasingly important for modern manufacturing systems [1]. As production lines grow in flexibility and complexity, robots are expected not only to execute predefined trajectories but also to provide feedback on their interactions with the environment [2]. Payload mass estimation is particularly valuable because the payload directly affects robot dynamics, operational safety, and process reliability. In industrial handling and assembly operations, the ability to verify whether an object is present, whether gripping was successful, or whether the transported part has the expected mass can significantly improve process reliability and quality assurance. Such information is usually obtained with dedicated external sensors, such as load cells or force–torque sensors. Although accurate, these devices add cost, require additional integration, and can reduce the robustness of the overall system [3]. In contrast, internal motor-current signals are already available within the control system and can therefore provide a low-cost basis for soft-sensing functions.

Recent research has therefore focused on extracting information from internal robot signals, particularly from motor currents, which inherently reflect the mechanical load acting on each axis. Current-based methods have already been applied to fault detection and condition monitoring in industrial robots, for example, in gearbox fault detection using drive current signals [4].

Furthermore, payload dynamics can be identified directly from motor-current signals, thereby avoiding uncertainties associated with torque conversion and enabling accurate weight estimation without external sensors [5]. In a related direction, payload identification based on parameter differences has been combined with nonlinear friction modeling, demonstrating the potential for real-time payload estimation in industrial robots [6].

In the presented work, payload estimation is addressed as a sensorless inference problem based on internal motor-current signals acquired directly from the robot controller. Instead of relying on external force–torque instrumentation or a detailed analytical model of the manipulator dynamics, the proposed approach treats actuator-related electrical signals as an indirect sensing source that carries information about the handled load. In this sense, the robot itself acts as a virtual sensor of the manipulated payload.

Regarding data processing, recent research and data-driven approaches in the field of signal analysis from robotic systems are based mostly on deep architectures, such as convolutional neural networks (CNNs), recurrent networks (LSTM/GRU), or other methods [7]. These architectures are useful when the model handles raw sequential data and is used to identify dependencies directly from the signal [8].

In the present work, however, data recorded using traces are transformed into compact statistical descriptors to enable fast data processing using MLP that can easily identify interactions between statistical current features while requiring less training data, lower computational complexity, and simpler interpretation than sequence-based architectures [9].

The study also investigates whether current signals from all six robot axes are sufficient for accurate payload estimation during a repeatable handling motion segment. Particular attention is paid not only to the estimation accuracy itself, but also to cross-robot generalization, because practical deployment requires the method to remain reliable across different physical units of the same robot type. The article, therefore, combines automated signal acquisition, feature-based processing, and data-driven regression into a single payload estimation pipeline validated on two industrial robots.

It should be noted that the algorithmic part of the study intentionally relies on a conventional feature-based MLP regression pipeline; therefore, the main contribution of this work lies primarily in the systematic engineering validation of controller-based current acquisition and in the quantitative analysis of cross-robot generalization, rather than in proposing a disruptive new machine learning architecture.

**Figure 1:**
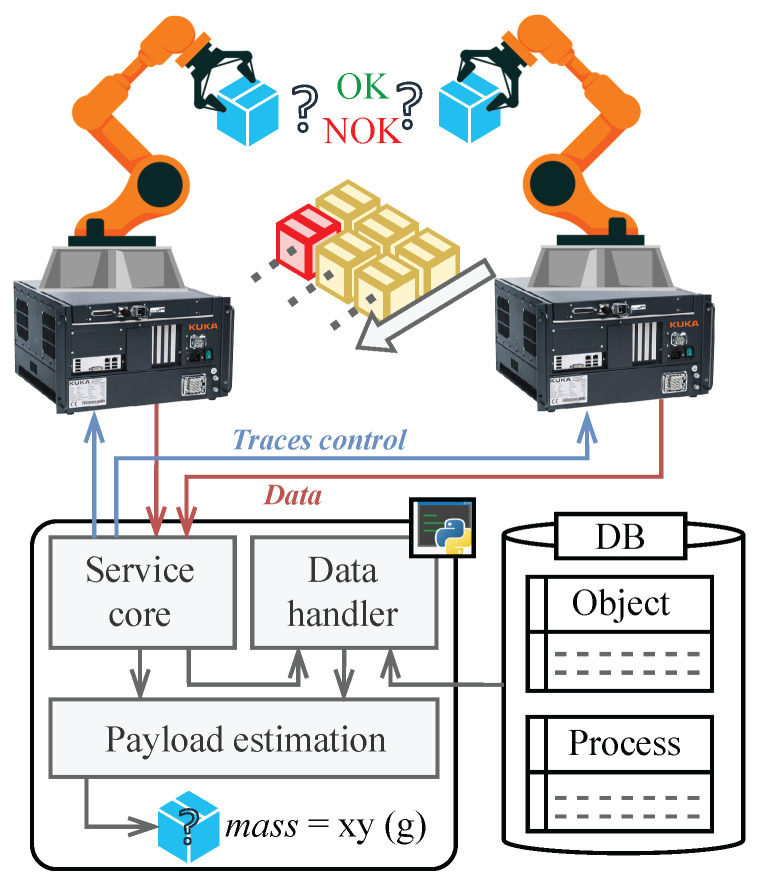
Conceptual diagram of the proposed payload estimation framework. Blue arrows indicate trace-control commands, red arrows represent the transfer of recorded motor-current data, and gray arrows show the internal data flow within the service application.

The core idea is illustrated in Figure 1. During the handling operation, the robots manipulate objects with different payload masses, while six-axis motor-current traces from each unit are recorded directly from the KUKA controllers. The acquired data are transferred to the service application, where they are processed by the data handler and passed to the payload estimation model, which is common for multiple units of the same robot type. The model outputs the estimated payload mass in grams, which can then be compared with predefined limits or expected values to support OK/NOK payload verification. The numerical mass estimate can also be used directly in subsequent process steps.

Recent studies have demonstrated that robot payload parameters can be identified from internal actuator signals without dedicated force or weight sensors. However, the majority of these approaches rely on an explicit dynamic model, prior identification of the unloaded robot, nonlinear friction compensation, or specially designed excitation trajectories. Furthermore, their experimental validation is generally focused on estimation accuracy within a single robotic system rather than on transferability across different physical units of the same robot type. In parallel, transfer learning and domain adaptation methods have been investigated in industrial robot diagnostics, but primarily for categorical fault diagnosis rather than continuous payload mass estimation.

To address these gaps, this study aims to validate whether internal six-axis motor-current signals support accurate cycle-level payload mass estimation and to quantify data-driven model generalization across two nominally identical industrial robot units. Furthermore, the following research objectives were defined: (i) To develop an automated procedure for acquiring synchronized six-axis motor-current traces directly from an industrial robot controller without additional measurement hardware. (ii) To develop a feature-based regression pipeline for estimating payload mass from statistical descriptors of the recorded current signals. (iii) To compare the same-robot estimation accuracy with direct cross-robot transfer between two nominally identical industrial robots. (iv) To determine whether mixed-source training using data from both robots reduces unit-specific estimation errors and improves generalization to unseen measurements.

This article is structured as follows: Section 2 reviews related work on sensor-based and sensorless payload estimation in industrial robots and cross-robot generalization. Section 3 describes the experimental setup, data acquisition workflow, dataset structure, feature extraction, and MLP-based regression model. Section 4 presents the payload estimation results, including architecture selection, cross-robot validation, and feature-importance analysis. Section 5 discusses the implications, limitations, and industrial applicability of the proposed approach, while Section 6 concludes the article, addresses the defined objectives, and outlines future work.

## 2. State of the Art

Research on payload estimation in industrial robots has developed along two principal directions: first, the use of dedicated external sensors, and second, sensorless approaches that exploit internal signals, such as motor currents and joint torques.

The traditional way to determine payload is based on force-to-torque (F/T) sensors or load cells mounted on the robot wrist or along the manipulator structure. These devices provide highly accurate measurements of external forces and moments, and their use is well established in tasks such as force-controlled assembly or precision handling. Nevertheless, their integration introduces several drawbacks, including increased hardware cost, the need for mechanical adaptation, and sensitivity to damage in industrial environments. Consequently, manufacturers and integrators often avoid using such sensors in large-scale production lines unless absolute accuracy is required [10].

To overcome the limitations of the traditional method, considerable effort has been invested in exploiting information already available within the robot control system. Because the electrical current of a drive motor is proportional to the torque produced, it inherently carries information about the external load. Several studies have therefore focused on estimating payload properties using dynamic models of the robot combined with current or torque measurements. Early work explored model-based identification techniques to infer inertial parameters of the payload, which can then be mapped to mass and center-of-gravity values [11]. More recently, current-driven methods have been applied in related fields such as fault detection and condition monitoring, showing that load variations and mechanical degradations can be detected reliably from drive signals [4]. Other research is showing that the torque difference between repeated, identical trajectories with and without the payload can be written directly as a function of the payload’s inertial parameters, removing the need for prior knowledge of link/rotor inertias [12].

Reliable sensorless payload estimation relies heavily on the quality and accessibility of motor-current data. In the literature, the acquisition of these signals from industrial manipulators is generally divided into two main paradigms: external hardware-based measurement and internal software-based extraction [13]. Multiple previous studies have focused on sensorless data acquisition from industrial robots, mostly for predictive maintenance [14]. For KUKA robots, a specialized library can be really helpful for data collection [15]. Moreover, KUKA controllers can be integrated through different communication and synchronization approaches, including low-cost network-based methods and external control interfaces, which makes automated trace configuration, triggering, and data transfer feasible [16,17].

Building on these foundations, innovative identification methods have been proposed that bypass the uncertainties of torque estimation and directly use raw current signals. For example, Tie et al. [5] demonstrated that analyzing current signals provides a more direct relationship with payload dynamics, thereby reducing modeling error. Xu et al. [6] further extended this principle with an online identification algorithm that incorporates nonlinear friction and parameter differences, enabling real-time estimation under varying operating conditions. These approaches underscore a broader research trend toward sensorless monitoring and adaptive control, in which industrial robots autonomously assess their operational state without external instrumentation.

However, traditional analytical and model-based methods heavily rely on accurate mathematical representations of robot dynamics, particularly of nonlinear joint friction, which is notoriously difficult to model and fluctuates with temperature, velocity, and mechanical wear [18]. To address these complexities, there is a growing transition toward data-driven and Machine Learning (ML) approaches. By employing architectures such as multilayer perceptrons (MLPs), researchers can bypass explicit physical modeling [19]. The algorithms implicitly learn the complex, nonlinear mapping between motor-current signatures and external payload mass directly from experimental data, offering a more flexible alternative to rigid mathematical models [20].

In robot and industrial signal analysis, deep learning has been applied through several architectural families. CNN-based models are commonly used to learn local patterns from transformed or windowed signals, recurrent models such as LSTM/GRU are used to represent temporal dependencies, and more recent transformer-family models are used for long-range dependencies in sequential data [7]. In industrial robot diagnostics, relevant research includes attention-based CNN models for reducer fault diagnosis and informer-based models for compound fault diagnosis [21,22].

Despite the advantages of data-driven models, a significant gap remains in the literature regarding their scalability and deployment across industrial fleets. Most current ML models are trained and validated on a single robotic unit. Nonetheless, mechanically identical robots exhibit unique electromechanical characteristics, such as distinct friction profiles, gear assembly tolerances, and current sensor zero-offsets, leading to substantial “unit-to-unit” variability. Consequently, a model trained on one robot often suffers a severe degradation in accuracy when deployed on another robot of the exact same make and model. This problem was also analyzed on daVinci Manipulators [23]. Overcoming this cross-robot generalization gap through multi-source training or transfer learning represents a critical next step for making sensorless estimation practically viable in large-scale manufacturing. Recent transfer learning studies in robotic and industrial systems show that knowledge learned from one platform, task, or operating condition can be reused to improve adaptation to new data distributions and reduce the need for complete model retraining [24,25]. In the context of the proposed method, such approaches could help compensate for robot-specific differences in motor-current signals while preserving the advantages of an external-sensor-free estimation architecture.

The issue of cross-robot generalization is closely related to transfer learning and domain adaptation. In robotics, transfer learning aims to reuse knowledge across different robots, tasks, or environments, while domain adaptation addresses the degradation that occurs when training and deployment data follow different distributions [24]. In industrial robot monitoring, this problem appears when models trained under one operating condition, process, machine, or robot unit are applied to another. Recent studies have therefore used adversarial domain adaptation for industrial robot joint-bearing fault diagnosis, transfer learning for servomotor bearing fault detection from current-derived scalogram images, and transfer learning-based cross-process fault diagnosis for industrial robots [26,27,28]. These studies show that adaptation strategies can reduce the need for full retraining. However, most of them address fault classification or process-condition transfer rather than continuous payload mass regression from internal motor-current features. The present work, therefore, positions cross-robot validation as one of its central experimental aims. It quantifies the unit-to-unit generalization gap between two nominally identical robots and evaluates mixed-source training as a practical first step toward more robust fleet-level deployment, as is also presented by Mahyari et al. [29].

### Recent Advances and Research Gap

Recent research relevant to sensorless payload estimation can be divided into three closely connected areas: controller-level signal acquisition, robot-specific payload and dynamic identification, and data-transfer methods addressing changes between robots, tasks, and operating conditions [14,24].

Xu et al. [30] developed a current-level dynamic-calibration framework in which robot dynamics were expressed directly in terms of motor currents, reducing errors associated with torque conversion. Their later iterative-weighting method further improved the identification of payload-induced current dynamics [11]. However, both approaches remain dependent on explicit robot dynamics and robot-specific identification, and neither evaluates continuous data-driven payload regression or transfer between physically distinct robot units.

In another study, Xu et al. [31] proposed a double-weighting method for identifying payload mass, center of mass, and inertial parameters. They subsequently introduced an online method based on differences between the dynamic parameters of the unloaded and loaded robot, including nonlinear friction modeling [6]. Although these methods improve analytical identification accuracy, they require an explicit parametrized model and prior robot-specific identification, while unit-to-unit generalization between nominally identical robots remains unexamined.

Recent work has also addressed payload-dependent adaptation. Shan and Pham [10] used model pre-training and a short calibration trajectory to adapt sensorless contact estimation to a new payload. The method reduces calibration effort, but its objective is contact estimation rather than direct payload mass regression, and transfer between different physical robot units was not quantified.

Research on domain adaptation confirms that robot-signal models are sensitive to differences between training and deployment conditions. Xia et al. [26] applied adversarial domain adaptation to robot-bearing diagnosis under changing loads and speeds, while Wang et al. [28] investigated transfer between different industrial processes. Yilmaz et al. [23] demonstrated the transfer of learned dynamics between different surgical robots and configurations. However, these studies address fault classification or external force estimation rather than continuous payload regression from controller–internal motor currents.

Kumar et al. [27] used transfer learning and current-derived scalogram images for servomotor-bearing fault detection. Although the study demonstrated that motor-current information can support transferable models, it addressed discrete fault classification and required time–frequency image conversion. It did not investigate numerical payload estimation or transfer between nominally identical industrial robot units.

In summary, the studies presented in Table 1 demonstrate that internal actuator signals are suitable for robot identification and monitoring, while distribution changes can substantially affect data-driven models. Nevertheless, three limitations remain. Firstly, recent payload identification methods are predominantly model-based and robot-specific. Secondly, transfer learning research primarily focuses on fault classification, contact estimation, and surgical-robot dynamics rather than continuous payload mass regression. Thirdly, little quantitative evidence exists on the performance degradation occurring when a payload estimation model is transferred between nominally identical industrial robots. This study addresses this gap by applying the same controller-based acquisition and regression pipeline to two KUKA KR3 units and comparing same-robot validation, direct cross-robot transfer, and mixed-source training.

## 3. Materials and Methods

This section describes the complete experimental and computational workflow used for payload mass estimation. It first introduces the data acquisition system based on internal motor-current traces from the robot controller, and then describes the experimental setup, payload configuration, and collected datasets. Finally, the machine learning pipeline used for feature extraction, regression modeling, and evaluation is presented.

### 3.1. Data Acquisition System

The experiments were conducted using two KUKA KR3 R540 six-axis industrial robots controlled by two KUKA KRC4 Compact controllers running KSS 8.5.8 and installed in a KUKA Ready2_educate cell (KUKA Deutschland GmbH, Augsburg, Germany). Motor-current data from all axes were acquired using KUKA Trace, an integrated diagnostic tool available in KUKA robot controllers. The NextGenDrive trace source and its Actual Current channel were used to record the motor currents, with each recording triggered by Motion Start. The data were sampled directly within the robot controller in its real-time VxWorks operating system, which is responsible for robot program execution, motion control, and the synchronization of all recorded signals. Consequently, all variables recorded within the same KUKA Trace share a common controller time base and are sampled equidistantly without being affected by network-induced latency or jitter. The sampling period was set to 12 ms, corresponding to a sampling frequency of approximately 83.3 Hz.

While traces can be configured and initiated manually via WorkVisual or the teach pendant, a dedicated Python (version 3.11) application was developed to automate trace configuration, triggering, data transfer, parsing, and logging. Trace recording was controlled through the system variable $TRACE, which was modified remotely over TCP/IP using the C3 Bridge Interface server running on the robot controller, and the py_openshowvar client running in the Python application on a PC. During each measurement, the trace data were stored in an internal buffer of the robot controller and transferred to the Python application only after the recording had been completed. The recorded files were downloaded via SFTP. Because KUKA Trace stores the recorded data in multiple .trc, .dat, and .r64 files, the Python application parsed these files and logged the extracted data into a single .csv file. Each resulting file contained seven columns: one column representing the sample number and six columns containing the actual motor-current values of the individual robot axes.

### 3.2. Experimental Setup

**Figure 2:**
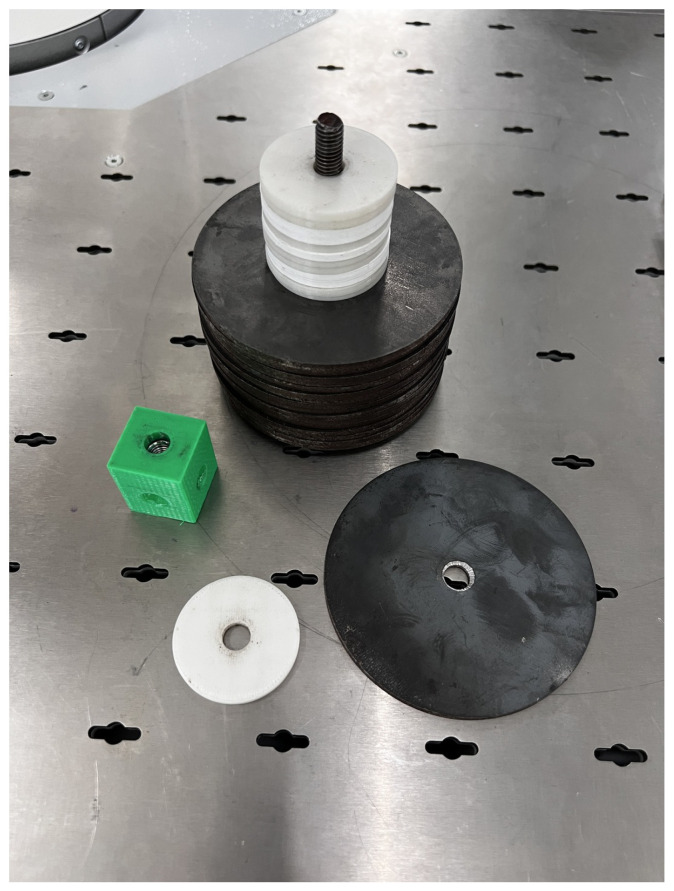
Adjustable weight fitting for robot load adjustments.

**Figure 3:**
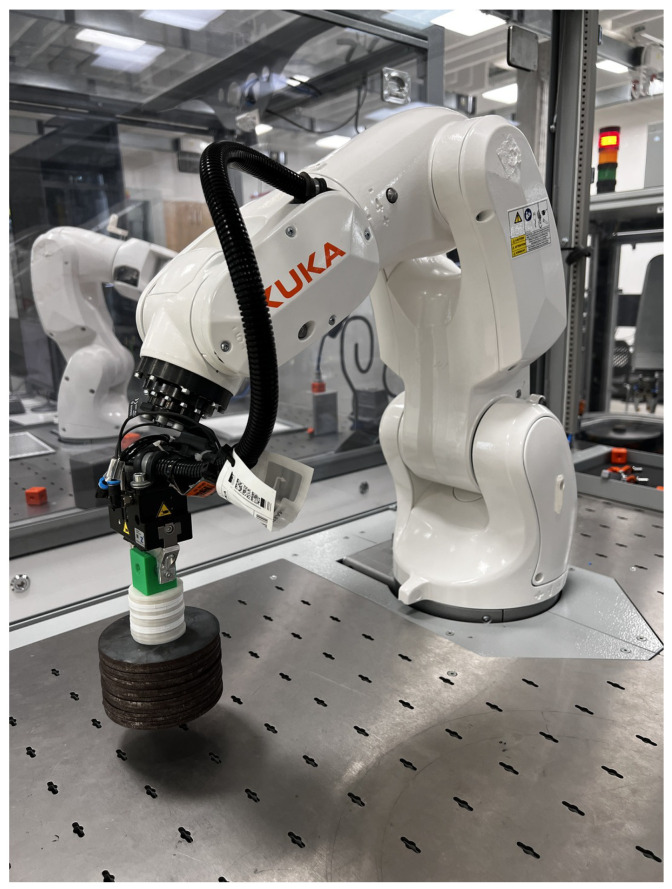
One of two KUKA KR3 R540 industrial robots in start position.

Every robot included in the experiment was equipped with a pneumatic gripper holding a custom payload fixture (Figure 2) composed of stackable metal discs, each weighing approximately 150 g. The stackable design allowed the payload mass to be varied from approximately 430 g to 2600 g in nominal 150 g increments, while keeping the geometry, gripping position, center of gravity, and inertia distribution approximately controlled. Because the individual discs had slightly different masses and the resulting increments were therefore nominal rather than exact, each assembled payload configuration was weighed on a scale before data acquisition, and its exact mass was carefully logged into the dataset file. In each test case, the robot executed five motion cycles, repeatedly moving axis A2 between $-75^{{}^{\circ}}$ and $-120^{{}^{\circ}}$, with the initial joint configuration set to A1–A6 = $\left[90^{{}^{\circ}},-75^{{}^{\circ}},105^{{}^{\circ}},0^{{}^{\circ}},30^{{}^{\circ}},0^{{}^{\circ}}\right]$, where A1 represents the first axis and A6 the sixth axis. This A2 movement was selected as a repeatable lifting-relevant segment of the handling cycle, because such a physically informative segment can be sufficient for the targeted payload-verification use case without necessarily evaluating the complete multi-axis trajectory. Figure 3 shows the setup, with the robot in its initial position and with the load in its gripper.

One dataset consists of motor current data for all 6 robot axes. For each dataset, the robot performed 5 movement iterations at 15% of the robot’s maximum speed. One iteration was moving the robot axis A2 from $-75^{\circ }$ to $-120^{\circ }$ and back. This axis reached a maximum speed of approximately $80^{\circ }$ per second. During data acquisition, only axis A2 was commanded to move. However, the remaining axes remained active, and their motors contributed to compensating for the dynamic effects induced by the axis A2 motion.

### 3.3. Dataset Construction, Sample Distribution, and Evaluation Strategy

**Figure 4:**
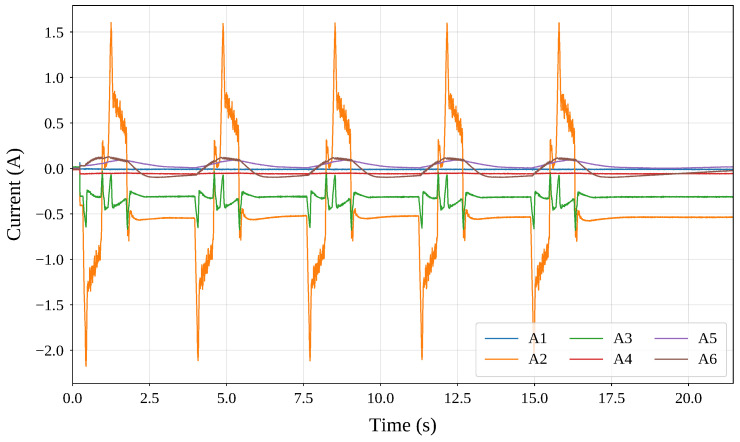
Motor-current signals of axes A1–A6 during repeated motion cycles with a payload of 437 g from dataset D1.

The list of the obtained datasets is provided in Table 2. Each dataset contains approximately 85,000 samples, with around 5300 samples recorded for each of the 16 measured payload masses. Figure 4 shows the motor-current signals recorded during one representative measurement from dataset D1 with a payload of 437 g. The displayed signals cover several repeated motion cycles. The largest current variations are observed for axis A2, which carries a substantial portion of the payload-induced load during the motion performed. The remaining axes exhibit smaller current variations corresponding to their coordinated motion and compensation of the payload and robot dynamics.

To evaluate the model on previously unseen recordings, the data were split at the dataset level using a Leave-Dataset-Out approach instead of a random sample-wise split. The motor-current signals within each recording form a continuous and temporally correlated sequence. Randomly assigning individual samples to the training and testing sets could, therefore, place closely related samples from the same recording in both subsets and lead to overly optimistic performance estimates. Three evaluation scenarios were considered: (a) same-robot generalization, in which the model was trained and tested on separate datasets acquired from the same physical robot; (b) direct cross-robot transfer, in which a model trained on one robot was evaluated on a nominally identical robot without fine-tuning or additional adaptation; and (c) mixed-robot training, in which data from both robots were combined to determine whether exposure to unit-specific variations improves generalization to unseen datasets.

### 3.4. Machine Learning

Machine learning methods were used to model the weight handled by the Kuka robot. The applied method was based on a pipeline with structured preprocessing and a feature-extraction phase, and a regression model based on a multilayer perceptron (MLP) neural network designed to capture complex, nonlinear patterns. The initial benchmarking between MLP, support vector machines (SVMs), random forest, and long short-term memory (LSTM) confirmed the superiority of MLP over other tested methods (Table 3).

The approach not only predicted the results but also analyzed the importance of the characteristics and influence of individual sensors. The aim of the learning pipeline was to use a transparent and reproducible data-driven baseline for validating whether internal motor-current traces can support payload estimation across different physical robot units, rather than introducing a novel neural-network architecture.

#### 3.4.1. Data Preprocessing and Feature Extraction

In the first stage of preprocessing, all non-numeric data and irrelevant data were removed from the dataset. Then, feature extraction was performed, which is a preferred method for raw sensor data that are too high-dimensional or noisy for direct modeling. For a dataset with *N* samples, where $x_{i,n}$ is the *n*-th sample of the *i*-th variable, the extracted metrics include the mean ($\mu_{i}=\frac{1}{N}\sum_{n=1}^{N}x_{i,n}$), minimum ($x_{i,\min}$), maximum ($x_{i,\max}$), median (${\tilde{x}}_{i}$), standard deviation ($\sigma_{i}=\sqrt{\frac{1}{N}\sum_{n=1}^{N}{\left(x_{i,n}-\mu_{i}\right)}^{2}}$), and signal energy ($E_{i}=\frac{1}{N}\sum_{n=1}^{N}x_{i,n}^{2}$). In the next stage, the datasets were split into training and testing subsets based on measurement identifiers. To ensure that all features contribute equally to the learning process and prevent features on larger scales from dominating optimization, a final standardization step was performed, in which the input features and target variables were transformed into *z*-scores by $z=\frac{x-\mu }{\sigma }$, where *x* is the raw variable and μ and σ are the mean and standard deviation, which are calculated from the training set.

#### 3.4.2. Regression Models Based on MLP Neural Networks

An MLP regression model was used to map the extracted features to the target weight. Although the input consists of statistical features extracted from motor-current signals, the relationship between these features and the payload mass remains highly nonlinear due to the combined effects of joint coupling, gravity compensation, friction, actuator dynamics, and controller behavior. Therefore, a nonlinear regression model based on a multilayer perceptron (MLP) was selected. The final network architecture was determined experimentally through a systematic hyperparameter search rather than being chosen a priori. The architecture consists of two hidden layers with $N_{h1}$ and $N_{h2}$ neurons, and each layer is followed by a rectified linear unit (ReLU) activation function to create the nonlinearity for complex estimation. The transformation performed by each layer *l* is defined as $h^{\left(l\right)}=f\left(W^{\left(l\right)}h^{\left(l-1\right)}+b^{\left(l\right)}\right)$, where $h^{\left(l-1\right)}$ is the input of the preceding layer, $W^{\left(l\right)}$ is the weight matrix, $b^{\left(l\right)}$ is the bias vector, and f(·) represents the ReLU function given by f(x)=max(0,x). The complete MLP performs a nonlinear mapping from the standardized input vector $x\in R^{d}$ to the predicted output $\hat{y}\in R$ through the following composition:

(1) $$\hat{y}=W^{\left(3\right)}f\;\left(W^{\left(2\right)}f\;\left(W^{\left(1\right)}x+b^{\left(1\right)}\right)+b^{\left(2\right)}\right)+b^{\left(3\right)}.$$

#### 3.4.3. Training and Optimization

To find the optimal model parameters $\left\{W^{\left(l\right)},b^{\left(l\right)}\right\}$, the mean squared error (MSE) loss between the predicted and actual weights ($L_{\mathrm{MSE}}=\frac{1}{N}\sum_{i=1}^{N}{\left(y_{i}-{\hat{y}}_{i}\right)}^{2}$) should be minimized. For optimization, the Adam algorithm was chosen with a learning rate of $\eta =10^{-3}$. The training cycle followed a standard iterative process of forward propagation (${\hat{y}}_{i}=f_{\theta }\left(x_{i}\right)$) and backward propagation ($\theta \leftarrow \theta -\eta \nabla_{\theta }L_{\mathrm{MSE}}$), where θ represents the collective trainable weights and biases of the MLP.

#### 3.4.4. Model Evaluation

After the training stage, the MLP’s performance on both the training and testing sets was evaluated for good generalization to unseen data. Several metrics were used to provide a multi-faceted view of predictive accuracy. These metrics included the mean absolute error (MAE) given by $\mathrm{MAE}=\frac{1}{N}\sum_{i=1}^{N}\left|y_{i}-{\hat{y}}_{i}\right|$ to provide a direct intuition of the average prediction error in grams, and the mean squared error (MSE) given by $\mathrm{MSE}=\frac{1}{N}\sum_{i=1}^{N}{\left(y_{i}-{\hat{y}}_{i}\right)}^{2}$ to show larger outliers more heavily and ensuring that the model remains stable across different weight classes and tolerance-based accuracy, as calculated using Equation (2), to provide the margins of error from the percentage of predictions falling within a defined tolerance δ, in the range of (δ=5,10,…,50 g) for the evaluation of the model’s reliability under varying precision requirements. In the final-stage evaluations, the results were visualized using scatter plots of predicted versus actual weights, which serve as a primary visual indicator of the model’s performance.

(2) $${\mathrm{Accuracy}}_{\pm \delta }=\frac{1}{N}\sum_{i=1}^{N}1\;\left\{|y_{i}-{\hat{y}}_{i}|\leq \delta \right\}\times 100\%.$$

#### 3.4.5. Permutation-Based Feature Importance

To better understand the resulting neural network and identify which sensor signals drive its predictions, a permutation importance analysis was performed to measure the importance of each feature by observing the model’s performance degradation when specific information is removed. For each feature $x_{j}$, the values in the samples were randomly shuffled to remove its predictive power while maintaining the overall distribution of data. The importance $I_{j}$ was then calculated as the average increase in the root mean square error (RMSE) over *R* shuffles using Equation (3), where ${\mathrm{RMSE}}_{j}^{\left(r\right)}$ is the error after the permutation feature *j* on repetition *r*, and ${\mathrm{RMSE}}_{\mathrm{baseline}}$ is the model’s original error on unshuffled data. Features that yield a higher $I_{j}$ are considered more influential; if shuffling a sensor’s data caused a spike in error, the model was relying heavily on that signal. This evaluation framework, which combines traditional regression metrics, tolerance-based accuracy, and feature importance, offers a comprehensive view of the model’s capabilities.

(3) $$I_{j}=\frac{1}{R}\sum_{r=1}^{R}\left({\mathrm{RMSE}}_{j}^{\left(r\right)}-{\mathrm{RMSE}}_{\mathrm{baseline}}\right).$$

## 4. Results

To identify the most effective MLP architecture for weight prediction in this study, an extensive grid search was performed on configurations of the hidden layers, which varied the number of neurons in both the first and second layers within the range of 8 to 1024 neurons. In total, 64 unique architectures were evaluated. Each iteration was trained on a consistent subset of data (datasets D1, D2, D3, D5, D6, and D7) and evaluated on the held-out datasets D4 and D8 for architecture comparison. The goal is to balance the model capacity with predictive stability, using the mean absolute error (MAE) and mean squared error (MSE) as primary benchmarks. Table 4 shows the top ten performing configurations and how various layer sizes impacted the overall precision of the robot’s weight estimation. To identify an appropriate model complexity, a systematic grid search was performed over 64 MLP architectures with hidden-layer sizes ranging from 8 to 1024 neurons. As shown in Table 4, the predictive performance generally improved with increasing model capacity until reaching the 512–512 configuration, which achieved the lowest testing MAE (6.75 g) and MSE (68.28 g^2^). Increasing the number of neurons beyond this point did not produce meaningful improvements in testing performance despite reducing the training error. Therefore, the 512–512 architecture was selected as the best compromise between predictive accuracy and generalization.

**Figure 5:**
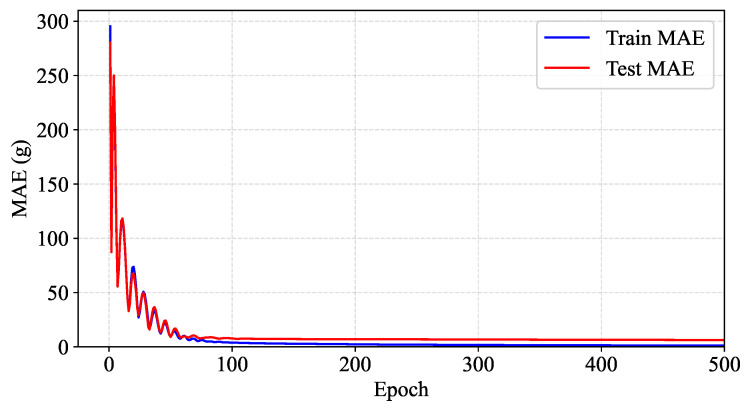
Mean absolute error (MAE) versus training epoch.

**Figure 6:**
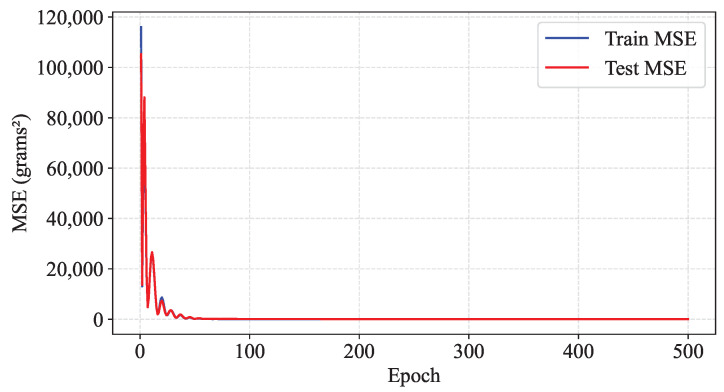
Mean squared error (MSE) versus training epoch.

The training behavior of the selected 512–512 architecture was examined over 500 epochs to assess convergence. Figure 5 and Figure 6 show the corresponding training and testing MAE and MSE curves. Both errors decreased rapidly during approximately the first 80–100 epochs and subsequently reached a stable plateau, with no clear divergence between the training and testing curves. For the experiments reported in this study, the model was trained for a fixed duration of 150 epochs, providing a sufficient margin beyond the observed convergence region while avoiding the additional computational cost of substantially longer training. The curves are presented only to illustrate the convergence behavior of the model and were not used as a validation-based early-stopping criterion. Because neither an independent validation subset nor an early-stopping procedure was employed, the selected training duration should not be interpreted as an independently optimized stopping point. This limitation should be addressed in future work using validation-based model selection and early stopping.

Using the selected 512–512 architecture and the fixed training duration of 150 epochs, the model’s performance across different combinations of training and evaluation datasets was examined. Table 5 shows the mean absolute error (MAE), mean squared error (MSE), and tolerance-based precision in multiple combinations of different datasets. The models trained and tested on data from the same robot achieved exceptional accuracy, with MAE and MSE values that are remarkably low, and high accuracy within the 15 g tolerance. However, the cross-robot generalization showed performance degradation. When data from Robot 1 (D1–D4) were applied to Robot 2 (D5–D8), the MAE increased beyond 80 g, and accuracy dropped below 15% within 15 g tolerance, which shows that mechanical or sensor calibration differences between individual units can significantly impact the model’s weight estimation. This gap was largely reduced by diversifying the training data. When the training set included samples from both robots, the model successfully learned a better representation, recovering to an accuracy of approximately 97% on the mixed testing data.

**Figure 7:**
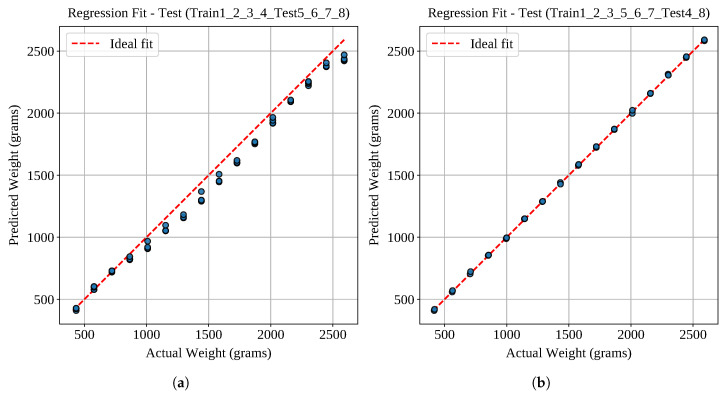
Regression results for the evaluated cross-robot generalization scenarios: (**a**) direct cross-robot transfer, with testing on D5–D8 after training on D1–D4; (**b**) mixed-source training, with testing on D4 and D8 after training on D1–D3 and D5–D7. Blue dots represent individual test sample predictions against actual weight values, while the dashed red line indicates the ideal fit.

Figure 7a shows the regression fit for the cross-robot test (trained with Robot 1, tested with Robot 2), revealing a strong linear correlation. However, a consistent downward shift from the identity line (y=x) indicates a systematic underestimation. This is a strong indicator of sensor calibration drift; the model correctly identifies the relationship between signal changes and weight. However, the absolute scaling or current offsets differ between the two robots. This indicated that, despite the same mechanical architecture, variations in electrical noise or factory calibrations necessitate cross-device normalization or calibration-tuning to achieve perfect transferability. On the other hand, Figure 7b illustrates using both robots for training and testing results in successful generalization with a very tight fit. This underscores that, for reliable deployment in fleet robotics, the inclusion of diverse hardware data is just as critical as the model architecture itself.

**Figure 8:**
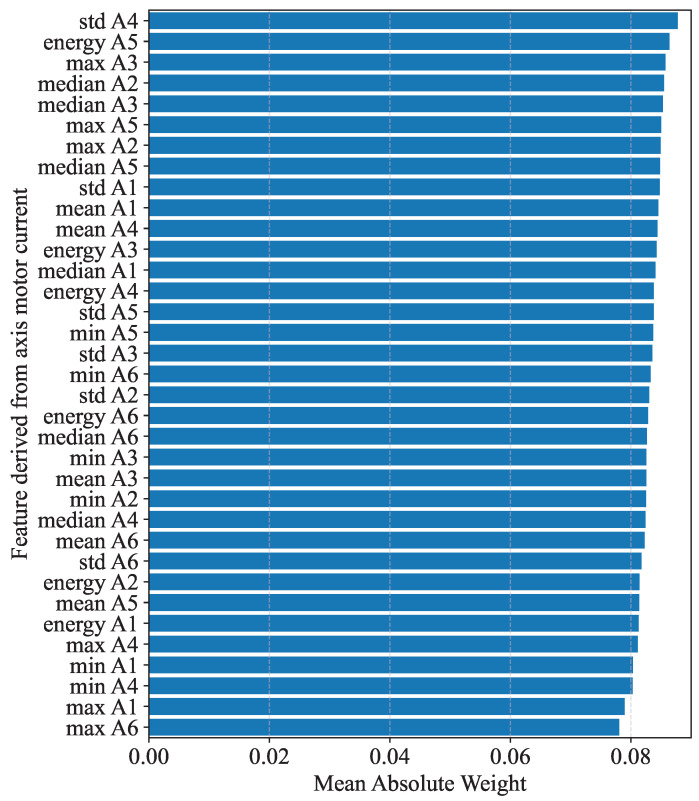
Feature importance for the model trained on D1–D3 and D5–D7, tested on D4 and D8.

For a better understanding of the model predictions, the feature weights were analyzed (Figure 8). At the level of individual extracted features, the standard deviation of the A4 motor-current signal showed the highest mean absolute weight, indicating that variations in this current signal carried strong information about the handled payload. Other highly ranked features were also derived from the currents of axes A2–A5. However, this feature-level result should not be interpreted as evidence that a single axis dominated the prediction, because individual feature importance does not directly represent the overall contribution of an entire robot axis.

**Figure 9:**
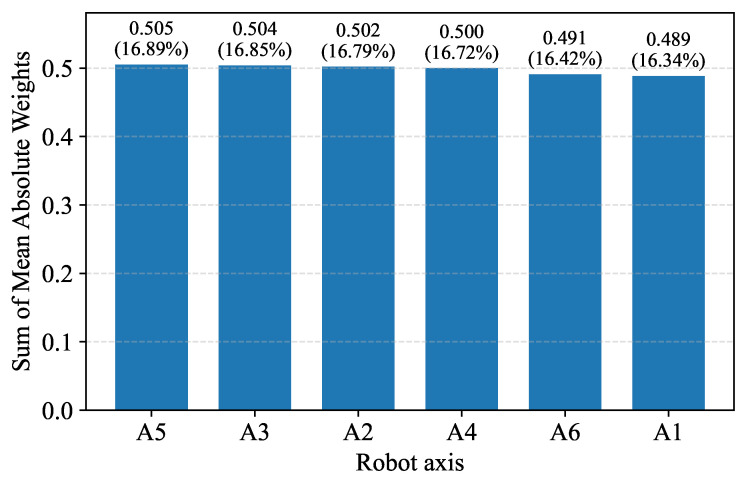
Aggregated feature importance by robot axis based on the summed mean absolute weights of all statistical current features.

The aggregated feature importance in Figure 9 provides a more axis-level view of the model behavior. After summing the contributions of all statistical features belonging to each axis, axes A5, A3, and A2 were identified as the most influential for the regression model. This is consistent with the experimental motion because these axes share the main plane of rotation in which the payload-induced excitation was performed. Therefore, although the standard deviation of the A4 current was the most important individual feature, the overall prediction relied more broadly on information distributed across axes A5, A3, and A2. In contrast, axes A1 and A6 showed the lowest aggregated importance, suggesting that payload variation had a less pronounced effect on their current signals during the tested motion.

However, the differences between the axes are not substantial. This suggests that the regression model did not rely on a single dominant joint, but rather used information distributed across several motor-current signals. This behavior is expected because the effect of an increased payload is propagated through the coupled robot kinematic chain and can influence the actuator currents of multiple axes, even if some axes are mechanically more exposed to the excitation than others.

## 5. Discussion

The results confirm that motor-current signals available directly from the robot controller contain sufficient information for payload estimation during a repeatable handling motion segment. The proposed approach differs from analytical payload identification methods in that it does not require an explicit dynamic model of the manipulator, detailed friction modeling, or external force/torque measurement. Instead, the payload mass is estimated from statistical features extracted from six-axis current traces. This makes the method attractive for industrial use, where additional sensors would add cost, complicate mechanical design, and increase maintenance demands. Its main application potential lies in payload verification, missing-part detection, wrong-part identification, unsuccessful gripping detection, or checking whether the handled object lies within an expected mass interval. In this context, regression provides a more flexible output than classification, because it gives a continuous estimate of mass that can be used both for OK/NOK decisions and for more detailed process monitoring.

The feature-importance analysis showed that payload-related information was not limited to the axis executing the main commanded motion. Although the experiment was based on the movement of axis A2, features derived from other active axes, especially A3–A5, also contributed strongly to the prediction. This behavior is expected because the payload affects the entire robot structure and the controller must compensate for gravity, posture-dependent dynamics, and mechanical coupling between joints. The result supports the decision to record current signals from all six axes instead of relying only on the primary moving joint.

The most important outcome of the study is the differences between same-robot accuracy and cross-robot transferability. When the model was trained and tested on the same physical unit, the prediction error was very low; for example, training on D5–D7 and testing on D8 resulted in an MAE of 2.60 g and 100% accuracy within the ±15 g tolerance. In contrast, direct transfer from Robot 1 to Robot 2 increased the MAE to 80.54 g and reduced the ±15 g accuracy to 14.60%. This result shows that nominally identical robots cannot automatically be treated as identical signal sources. Differences in friction, drive calibration, current offsets, gearbox condition, mechanical tolerances, temperature, or wear can shift the current signatures enough to degrade a model trained on another unit. The cross-robot regression results indicate that the model still captured part of the payload-related trend, but the absolute calibration was no longer valid. This points to a practical direction for improvement: cross-robot deployment will likely require at least a short calibration, normalization step, or transfer-learning procedure. The sensitivity of the model to varying payload shape, gripping position, center-of-mass shifts, and inertia distribution remains to be validated in future work.

The mixed-source experiment confirms this interpretation. When data from both robots were included in the training set, the MAE on unseen datasets decreased to 5.37 g, and the accuracy within the ±15 g tolerance reached 96.88%. The improvement indicates that the current signals do contain transferable payload information, but this information is partly masked by robot-specific characteristics. For deployment in production environments, this means that training on a single robot is probably insufficient unless the model is calibrated for each new unit. A more robust strategy is to collect representative data from several robots of the same type, or to introduce a lightweight reference-payload calibration step before using the model for monitoring.

Beyond the evaluated cross-robot generalization, future work should also address generalization across different robot motions. The present study deliberately used a repeatable handling trajectory to isolate the influence of payload mass and robot-specific signal differences. In practical applications such as palletizing, depalletizing, machine tending, or repetitive pick-and-place operations, the robot performs multiple trajectories with different poses, velocities, and acceleration profiles. The proposed approach should therefore be extended and validated across a broader set of motion patterns to enable the model to separate payload-related current changes from motion-related effects. This would support the more flexible deployment of sensorless payload estimation in real industrial handling tasks.

Although feature extraction and MLP inference are computationally lightweight once the trace data are available, the current implementation downloads the KUKA Trace data only after the motion segment has been completed. Therefore, the method does not provide hard real-time feedback during robot motion. This does not necessarily limit practical use in cycle-level payload verification, because the system can inform the operator or higher-level production logic immediately after the handling segment, or after the completion of a palletizing sequence, if any transferred object had a mass different from the expected value. In the current implementation, the required signal window corresponds to one completed selected motion segment used for feature extraction, namely the recorded A2 lifting-like segment sampled at a 12 ms interval.

Several limitations define the scope of the presented results. The experiments were performed on two robots of the same type and controller family, which is sufficient to demonstrate unit-to-unit variability but not enough to describe its distribution across a larger fleet. The model was also validated only for one predefined lifting-like motion segment, one speed setting, and a controlled payload fixture selected to isolate the influence of payload mass. Because motor currents depend on the trajectory, velocity, acceleration, robot posture, friction state, temperature, payload inertia, and center-of-gravity position, the present model should be considered motion-specific. The tested payloads mainly differed in mass, while their geometry, gripping position, approximate center of gravity, and inertia distribution were intentionally kept controlled. Consequently, the direct transfer of the model to complex multi-axis pick-and-place trajectories or to payloads with varying shape, center of mass, and inertia has not yet been validated. Finally, the current implementation validates the estimation principle using recorded KUKA Trace segments and subsequent feature extraction. Future work should therefore focus on broader validation across trajectories, speeds, payload geometries, robot units, and long-term operating conditions, with particular attention to cross-robot calibration and transferability.

For long-term industrial deployment, the model should also account for slow changes in the robot’s current response caused by mechanical wear, thermal drift, gearbox lubrication changes, or sensor offset variations. The current implementation does not explicitly separate these effects from payload-induced current changes; therefore, periodic recalibration would be required in practical use. A feasible strategy is to perform an automated reference-payload calibration at the beginning of each shift, using a known calibration mass handled by the robot, and to retrain or update the model after a defined operating interval. If the required calibration correction exceeds a predefined threshold, this deviation could also be used as an indirect indicator of changing robot condition and support condition-monitoring functions.

## 6. Conclusions

This article presents a sensorless method for cycle-level payload mass estimation in an industrial robot using only internal motor-current signals. The proposed approach combines automated trace acquisition, statistical feature extraction, and MLP-based regression to estimate the manipulated mass without external sensors or explicit dynamic modeling. Experimental validation on two KUKA KR3 robots showed that the method can achieve high accuracy under controlled, repeated-motion conditions, particularly when the training data include samples from both robotic units.

In line with the stated research aim and objectives, the principal contributions of this study are as follows:

(i) A controller-native payload estimation workflow was developed using only synchronized six-axis motor-current traces, without an external sensor.
(ii) A feature-based MLP regression pipeline was experimentally validated for cycle-level payload estimation over a payload range of approximately 0.4–2.6 kg.
(iii) The unit-to-unit generalization gap was quantified experimentally. Direct transfer from Robot 1 to Robot 2 increased the testing MAE to 80.54 g and reduced accuracy within ±15 g to 14.60%, despite the robots being nominally identical. Mixed-source training was shown to mitigate robot-specific signal differences, reducing the testing MAE to 5.37 g and increasing accuracy within ±15 g to 96.88%.
(iv) Feature analysis showed that the payload information was distributed across several robot-axis current signals rather than being confined to the commanded axis.

These findings indicate that internal motor currents can provide accurate payload information under repeatable operating conditions, but they also show that models trained on only one physical robot should not be assumed to transfer directly to another unit without calibration, normalization, adaptation, or representative multi-robot training.

The results also reveal an important practical limitation of single-source learning: a model trained on one robot does not transfer reliably to another nominally identical unit without a substantial loss of accuracy. This finding highlights that robot-specific electromechanical differences must be considered when designing data-driven estimation systems for industrial deployment. Within the evaluated motion-specific setting, the proposed method can support cycle-level verification of whether the handled payload lies within an expected mass interval. Its application for gripping validation, missing-part detection, and broader handling or assembly tasks remains a potential use case that requires validation across additional trajectories, payload configurations, and operating conditions.

Future work will focus on extending the method to a wider range of trajectories, increasing robustness across additional robotic units, and evaluating alternative learning strategies that reduce the amount of data required for adaptation to a new robot. Another promising direction is the integration of the estimation model into higher-level production logic, where payload feedback could be used for adaptive handling, process monitoring, and predictive maintenance.

## Figures and Tables

**Table 1:** Critical comparison of recent studies related to the identified research gap.

Study	Main Approach	Limitation Relative to the Present Study
Izagirre et al. (2021) [14]	Synchronized data-acquisition network architecture for predictive maintenance of industrial robots in manufacturing assembly lines.	The study focused on data-acquisition infrastructure, communication, and signal synchronization. Payload estimation, further processing, or generalization is missing.
Xu et al. (2022) [30]	Current-level robot dynamic calibration using internal motor-current signals.	The method requires an explicit robot dynamic model and robot-specific parameter identification. It was evaluated on a single UR10 robot without payload mass regression or cross-unit validation.
Xu et al. (2022) [31]	Double-weighting method for identifying payload inertial parameters.	The study focused on analytical identification accuracy rather than the transferability of a data-driven payload estimation model between physical robot units.
Xia et al. (2022) [26]	Adversarial domain adaptation for industrial robot joint-bearing fault diagnosis under varying operating conditions.	The method addressed categorical fault classification using vibration signals, not continuous payload mass estimation from controller–internal motor-current signals.
Xu et al. (2024) [6]	Online payload identification based on parameter differences and nonlinear friction modeling.	The method requires prior identification of the unloaded robot and an explicit parametrised dynamic model. Cross-robot transfer between separate physical units was not evaluated.
Kumar et al. (2024) [27]	Transfer learning for servomotor-bearing fault detection using motor-current scalograms.	The study addressed bearing-fault classification and required time–frequency image transformation. Continuous payload regression and same-type cross-robot transfer were not investigated.
Wang et al. (2024) [28]	Domain-adaptive cross-process fault diagnosis transferring knowledge between different operating processes of industrial robots.	The method addressed categorical fault classification across process conditions rather than robot-to-robot transfer or continuous payload mass regression using motor-current features.
Shan and Pham (2024) [10]	Pre-trained neural-network models combined with a short online trajectory for payload-dependent sensorless contact estimation.	The method requires payload-specific online calibration and targets contact estimation rather than direct payload mass regression. Transfer between different physical robot units was not quantified.
Xu et al. (2025) [11]	Identification of payload-induced current dynamics using iterative weighting estimation.	The study focused on current-level dynamic identification and reducing errors associated with torque conversion. It did not evaluate lightweight feature-based regression or transfer between nominally identical robot units.
Luo et al. (2025) [20]	Sensorless payload estimation using an improved disturbance Kalman filter with a variable-parameter noise model.	The method is based on a model-dependent observer and does not evaluate feature-based learning from six-axis controller currents or transfer between nominally identical industrial robot units.
Present study	Feature-based MLP regression using synchronized six-axis motor-current traces acquired from two nominally identical industrial robot controllers.	The study quantifies the unit-to-unit generalization gap in continuous payload mass estimation and evaluates mixed-source training as a strategy for reducing robot-specific estimation errors.

**Table 2:** Distribution of samples across the payload measurements for robotic units R1 and R2.

Robot R1							Robot R2					
Weight (g)	D1	D2	D3	D4	Total		Weight (g)	D5	D6	D7	D8	Total
437	5364	5347	5352	5348	21,411		434	5366	5365	5348	5350	21,429
582	5363	5349	5347	5350	21,409		579	5367	5350	5349	5351	21,417
727	5367	5348	5350	5349	21,414		724	5364	5350	5352	5349	21,415
872	5366	5347	5329	5349	21,391		869	5364	5350	5348	5369	21,431
1017	5471	5349	5350	5349	21,519		1014	5367	5352	5352	5351	21,422
1163	5366	5352	5351	5352	21,421		1160	5367	5352	5351	5352	21,422
1307	5363	5350	5349	5353	21,415		1305	5351	5352	5350	5349	21,402
1451	5365	5348	5346	5347	21,406		1450	5362	5354	5357	5352	21,425
1597	5364	5348	5352	5350	21,414		1594	5367	5347	5349	5352	21,415
1739	5365	5350	5351	5350	21,416		1739	5365	5350	5353	5349	21,417
1884	5365	5351	5351	5348	21,415		1884	5366	5347	5352	5348	21,413
2029	5363	5347	5349	5362	21,421		2030	5362	5351	5350	5348	21,411
2174	5362	5354	5348	5352	21,416		2175	5364	5350	5349	5351	21,414
2317	5364	5350	5347	5353	21,414		2319	5364	5349	5352	5347	21,412
2463	5363	5349	5352	5349	21,413		2464	5367	5352	5350	5350	21,419
2609	5364	5367	5348	5347	21,426		2609	5366	5350	5349	5350	21,415
Total	85,935	85,606	85,572	85,608	342,721		Total	85,829	85,621	85,611	85,618	342,679

**Table 3:** Model comparison performance metrics with MSE.

Model	MAE	RMSE	MSE	Accuracy (±50 g) (%)
Regression (MLP)	11.13	14.038	197.06	100.00
SVM	11.51	15.97	254.90	100.00
Random Forest	28.47	34.84	1214.10	85.71
LSTM	35.13	43.29	1874.17	85.71

**Table 4:** Top 10 MLP architectures from the grid search ranked by test MAE.

Hidden_1_	Hidden_2_	Training				Testing		
		MAE [g]	RMSE [g]	MSE [g^2^]		MAE [g]	RMSE [g]	MSE [g^2^]
512	512	3.60	4.83	23.30		6.75	8.26	68.28
1024	512	2.65	3.39	11.47		6.79	8.56	73.25
512	1024	3.66	4.66	21.73		6.76	9.04	81.68
1024	256	3.13	4.16	17.31		6.83	8.50	72.19
1024	128	3.33	4.53	20.52		7.36	9.13	83.33
1024	64	4.26	5.26	27.72		7.71	9.16	83.93
128	512	6.34	8.35	69.68		8.65	10.73	115.08
256	1024	4.80	5.98	35.71		8.34	10.19	103.91
512	256	4.53	6.06	36.72		8.51	11.43	130.71
1024	32	5.81	7.34	53.88		10.17	11.92	142.20

**Table 5:** Model performance across different training and testing dataset combinations using the best MLP configuration (512–512, 150 epochs). Metrics include mean absolute error (MAE), root mean square error (RMSE), mean squared error (MSE), and accuracy within ±15 g tolerance.

Training						Testing				
Dataset(s)	MAE [g]	RMSE [g]	MSE [g^2^]	Acc_±15_ [%]		Dataset(s)	MAE [g]	RMSE [g]	MSE [g^2^]	Acc_±15_ [%]
D1, D2, D3	2.31	3.42	11.69	100.00		D4	13.37	16.19	262.06	62.50
D5, D6, D7	2.08	2.94	8.65	100.00		D8	2.60	3.39	11.48	100.00
D1, D2, D3, D5, D6, D7	2.45	3.38	11.45	100.00		D4, D8	5.37	6.78	45.93	96.88
D1, D2, D3, D4	2.39	3.21	10.30	100.00		D5, D6, D7, D8	80.54	93.87	8810.67	14.60

## Data Availability

The experimental motor-current dataset supporting the findings of this study is openly available on Zenodo at https://doi.org/10.5281/zenodo.21456276.

## Abbreviations

Acc	Accuracy
CNN	Convolutional neural network
GRU	Gated recurrent unit
F/T	Force–torque
KSS	KUKA system software
MLP	Multilayer perceptron
MSE	Mean squared error
MAE	Mean absolute error
LSTM	Long short-term memory
NOK	Not OK/non-conforming state
OK	Correct/acceptable state
SFTP	Secure file transfer protocol
SVM	Support vector machine
RMSE	Root mean square error
RTOS	Real-time operating system
STD	Standard deviation
